# Heterogeneity of Phenotype and Function Reflects the Multistage Development of T Follicular Helper Cells

**DOI:** 10.3389/fimmu.2017.00489

**Published:** 2017-04-28

**Authors:** Marta Trüb, Tom A. Barr, Vicky L. Morrison, Sheila Brown, Stefano Caserta, Jordan Rixon, Alasdair Ivens, David Gray

**Affiliations:** ^1^Institute of Immunology and Infection Research and Centre for Immunity, Infection and Evolution, School of Biological Sciences, University of Edinburgh, Edinburgh, UK; ^2^Institute of Infection, Immunity and Inflammation, University of Glasgow, Glasgow, UK; ^3^Manchester Collaborative Centre for Inflammation Research, University of Manchester, Manchester, UK; ^4^Brighton and Sussex Medical School, University of Sussex, Brighton, UK; ^5^Graduate Group in Immunology, University of California Davis, Davis, CA, USA

**Keywords:** T follicular helper cells, T cell memory, T follicular helper cell subsets, germinal centres, T cell heterogeneity

## Abstract

T follicular helper cells (Tfh) provide crucial signals for germinal center (GC) formation, but Tfh populations are heterogeneous. While PD1^hi^ Tfh are important in the GC response, the function of the PD1^lo^ Tfh-like subset is unknown. We show that these cells, like the PD1^hi^ GC–Tfh, depend upon B cells; however, their entry to follicles is independent of CXCR5 or cognate interactions with B cells. The differentiation into PD1^hi^ Tfh is dependent on MHC class II interactions with B cells and requires CXCR5. Our data suggest a Tfh differentiation pathway that is initially B cell-independent, then dependent on non-cognate B cell interactions, and finally following cognate interaction with B cells and CXCR5-ligands allows the formation of GC–Tfh. The PD1^lo^ Tfh-like cells make early cytokine responses and may represent precursors of CD4 memory cells.

## Introduction

The segregation of B and T cells into separate compartments of secondary lymphoid organs allows for their critical interactions to occur under specialized conditions. This organization may enhance these interactions, but potentially also control their outcome and magnitude. There are at least two recognized sites of B–T cell interaction: the first is at the border between the T zone and the B cell follicle (1–3) and the other is within the follicle itself as a prelude to, and as part of, the germinal center (GC) reaction (4–6). The migration of T cells to the follicular border and the subsequent entry of some of these T cells into follicles are thought to be caused by balancing the expression levels of chemokine receptors CCR7 and CXCR5 (7). It has become axiomatic that the T cells that enter follicles do so because they are specialized helpers of B cells, and these have become known as T follicular helper cells (Tfh). Tfh are critical for the formation and function of GCs to bring about the generation of memory B cells and long-lived plasma cells [reviewed by Vinuesa et al. (8)]. However, some Tfh function is directed at extrafollicular B cell responses (9) and there is evidence of complexity in the subsets of Tfh, with some follicular T cells having regulatory function and phenotype (10) and others belonging to the iNKT subset (11). In this study, we investigate further the potential heterogeneity within the CD4 T cell populations that are currently grouped under the umbrella of Tfh.

T follicular helper cell differentiation programme is initiated upon the contact with cognate dendritic cell (DC) in appropriate cytokine milieu and in the presence of costimulatory molecules, of which ICOS is of particular importance (12). Activated precursors of Tfh (pre-Tfh) upregulate expression of CXCR5, ICOS, PD-1, and Bcl6 while downregulating CCR7 on the surface (8, 13). As a consequence, pre-Tfh migrate to the T–B cell border where the second step of Tfh differentiation occurs. Upon contact with cognate B cells, pre-Tfh stabilize and further enhance the expression of CXCR5, Bcl6, and PD-1 and become mature GC–Tfh, upregulating markers such as GL7 while expressing low levels of CCR7, IL-7R, and PSGL-1 on their surface (8). Many previous studies have shown that the presence of cognate B cells is required for ultimate completion of Tfh differentiation (2, 14–17).

Our previous work has highlighted the fact that B–T cell interaction, in general, comprises signals passing in both directions, beyond delivering help to B cells, T cells also receive signals from B cells in the form of antigen presentation, costimulation, and cytokines, all of which influence the programme of T cell differentiation (18, 19). In fact, this critical dialog is most clearly demonstrated by the dependence of Tfh on the receipt of B cell-derived signals (7, 17, 20), most importantly ICOS ligation (21). Our studies on the development of CD4 T cell memory have shown very clearly for several antigens and bacterial infection that, in the absence of antigen presentation by B cells, there is an almost complete loss of memory responses (18, 19, 22). Other labs have seen similar dependencies (23) in responses to viruses (24) and parasites (25, 26). We have been struck by the parallels between CD4 T cell memory development and conditions required to sustain Tfh; for instance, as well as a B cell dependency there also seems to be a requirement for expression of Bcl-6 in T cell memory precursors (27, 28) as there is for Tfh development (20, 29, 30). Thus, we are intrigued by the possibility that many of the T cells that enter B cell follicles may not differentiate to become GC-supporting Tfh, but perform other roles or pass into the memory pool.

Our interest in this possibility was sparked by the observation, described here, that Tfh generation during *Salmonella* infection occurred quite early (peaking at 7 days postinfection), but was unconnected to GC formation, which happened much later following infection (around 50 days). Despite this, and apart from expression of intermediate levels of PD1, the *Salmonella* Tfh cells had all the hallmarks of Tfh in that they expressed Bcl-6 and were dependent upon B cells for their survival. This PD1^lo^ Tfh-like population is readily apparent following most types of immunizations (e.g., with sheep red blood cells, SRBC), and so, we set out in this study to investigate the differentiation of PD1^lo^ Tfh-like cells, their relationship to PD1^hi^ Tfh, and also their function. Using a variety of bone marrow (BM) chimeric mice, we have defined multiple steps in the Tfh-differentiation pathway that have distinct molecular requirements. The PD1^lo^ Tfh-like population can give rise to GC-supporting PD1^hi^ Tfh, but, importantly, also have functionality of their own.

## Results

### Characteristics of the Tfh Response during *Salmonella* Infection

**Figure 1:**
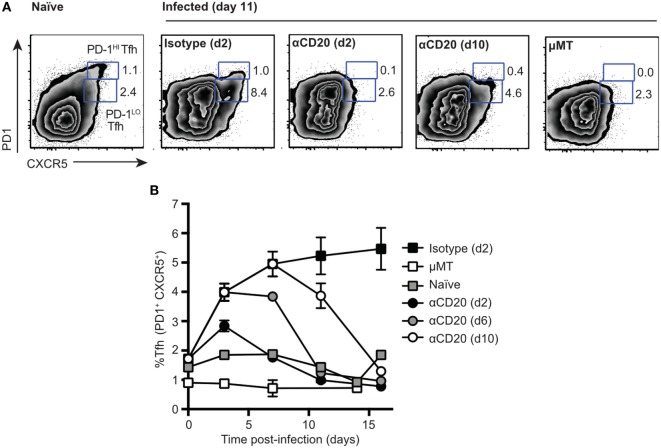
**Two populations of T follicular helper cells (Tfh), PD1^lo^ and PD1^hi^, are both dependent on B cells**. C57Bl/6 and μMT mice were infected with *Salmonella* (strain SL3261) and then treated with anti-CD20 at 2, 6, or 10 days postinfection. Control mice included infected mice receiving an injection of isotype control antibody at day 2 postimmunization and uninfected, naïve mice. **(A)** Representative FACS plots of Tfh staining (CXCR5 versus PD1) at day 11 postimmunization. Numbers in gates represent frequency among CD4 of PD1^hi^ and PD1^lo^ Tfh-like cells. **(B)** Time-course of Tfh expansion and contraction following B cell depletion with anti-CD20. Each data point represents the mean value for *n* = 5, and the bars SEM. Data are representative of three independent experiments.

Following intravenous infection with *Salmonella*, despite disruption of splenic lymphoid architecture, the number of CD4^+^ T cells inhabiting spleen B cell follicles increased (Figure S1A in Supplementary Material). As assessed by flow cytometry, these cells expressed the typical Tfh cell markers CXCR5, PD1 (Figure S1A in Supplementary Material), and ICOS (not shown). The kinetics of Tfh appearance was similar to that noted for SRBC antigens, peaking in the first week and declining slowly thereafter (Figure S1B in Supplementary Material). Interestingly, during *Salmonella* infection, almost all Tfh cells expressed intermediate to low levels of PD1 (PD1^lo^ Tfh-like cells), while the PD1^hi^ population seen in the response to SRBC was largely missing (Figure S1 in Supplementary Material). This is in contrast to SRBC immunization, where both populations of PD-1^lo^ Tfh-like cells and PD-1^hi^ Tfh are formed within first 7 days p.i. (Figure S1C in Supplementary Material). To investigate whether the PD1^lo^ Tfh-like cells generated in response to *Salmonella* were dependent on B cells [as previously shown for PD1^hi^ Tfh responses (7, 20, 21)], we depleted mice of B cells using anti-CD20 monoclonal antibody injections at different times postinfection (Figure 1). B cell depletion at day 2 and day 6 (Figure 1B) postinfection caused the complete loss of PD1^lo^ Tfh-like populations by day 11 (Figure 1A). Mice depleted of B cells at day 10 postinfection showed a partial loss of both Tfh populations by day 11 (Figure 1A). However, by day 16 postinfection, Tfh cells further decreased to background levels (1–2%) as detected in mice genetically deficient of B cells (μMT) (Figure 1B). These data demonstrate clearly that the PD1^lo^ Tfh-like cells generated after *Salmonella* infection are also exquisitely dependent on the presence of B cells for their continued survival (Figure 1B).

**Figure 2:**
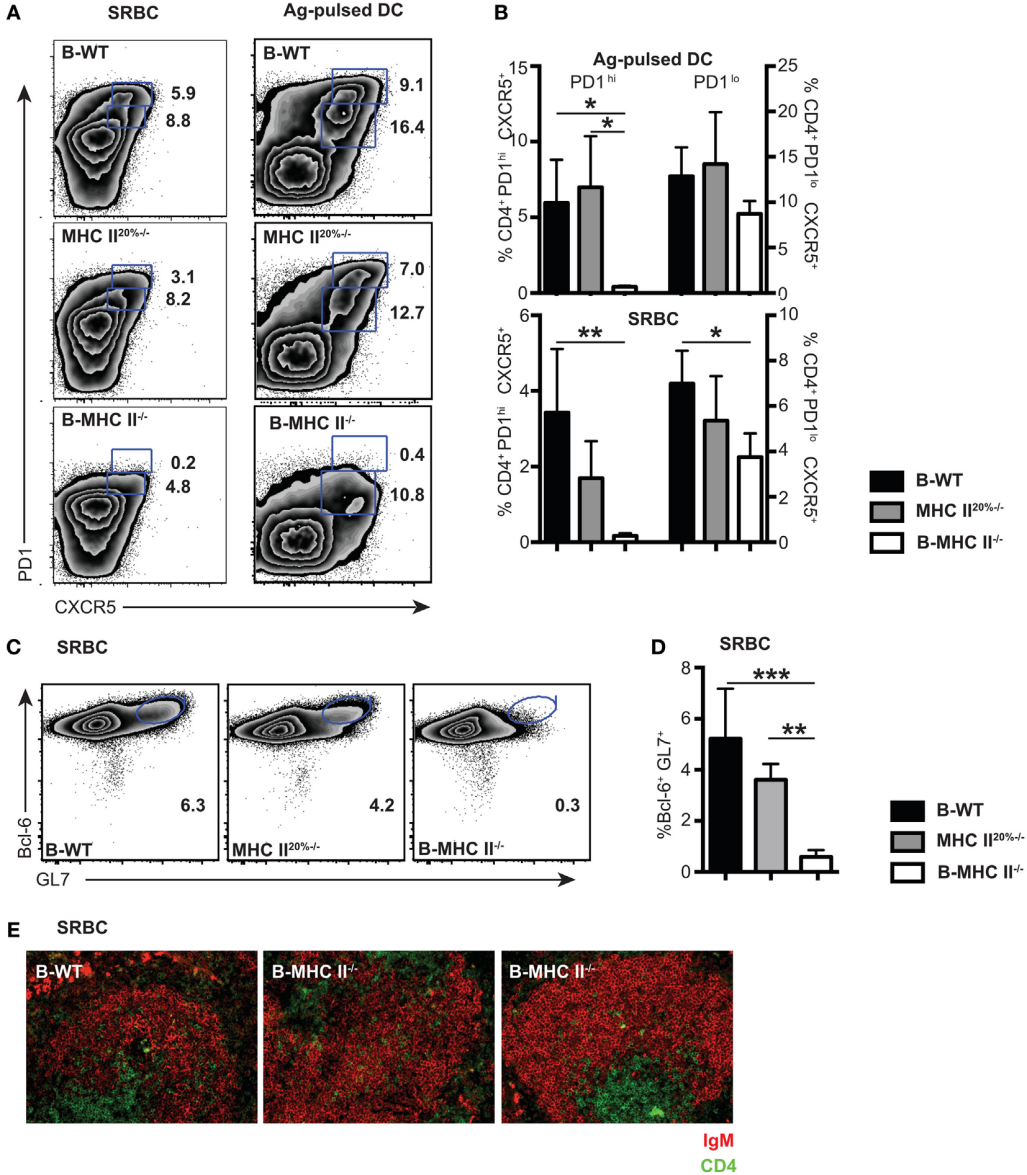
**The role of cognate interactions with B cells for the development of PD1^hi^ and PD1^lo^ T follicular helper cells (Tfh)-like**. Mixed-BM chimeras in which B cells do not express MHC II (B-MHC II^−/−^) or control (B-WT and MHC II^20%−/−^) were immunized with SRBCs or Ag-pulsed BM-derived dendritic cells. Tfh frequency and phenotype was assessed 7 days postimmunization by flow cytometry for CXCR5 and PD1 expression. **(A)** Representative FACS plots gated on CD4^+^ T cells show PD1^hi^ and PD1^lo^ Tfh-like populations generated following each immunization. **(B)** Mean frequencies of Tfh among CD4 T cells for each immunization on groups of five mice. Germinal centers (GCs) were also enumerated in SRBC immunizations at day 7 by flow cytometry staining of B cells for GL-7 and Bcl-6. Panel **(C)** shows representative FACS plots gated on CD19^+^ B cells, and panel **(D)** shows mean GC B cell frequencies among CD19^+^ splenocytes. **(E)** Pictures show immunohistochemical staining of splenic sections from SRBC immunized mice for B cell (IgM: red) and T cell (CD4: green) markers. T cell infiltration of B cell follicles can clearly be seen in all three chimeras. Data presented are representative from three (SRBC) and two [Ag-pulsed dendritic cell (DC)] independent experiments. Bar graphs show mean values for *n* = 4, with error bars SEM. Statistical analysis is by one-way ANOVA where *p < 0.05, **p < 0.01, and ***p < 0.005.

It is important to recognize that the Tfh response to a classical antigen, such as SRBC and antigen-pulsed DC, consists of both PD1^lo^ and PD1^hi^ populations at day 7 after immunization (Figure 2A, Figure S1A in Supplementary Material). The PD1^lo^ Tfh-like population often dominated over PD1^hi^ Tfh cells at relatively early time points, accounting for up to >60% of Tfh cells, especially seen in the case of antigen-pulsed DC (Figure 2A). Having established that the PD1^lo^ Tfh-like are, similar to PD1^hi^ Tfh, B cell dependent, we wished to know whether these two populations had similar differentiation requirements and whether they had a precursor–product relationship.

### Requirements for Cognate Interactions with B Cells of PD1^hi^ and PD1^lo^ Tfh Like

We first investigated whether PD1^hi^ and PD1^lo^ Tfh-like populations had similar requirements for antigen presentation by B cells. The generation of PD1^hi^ Tfh is initiated by antigen presentation from DC (21, 31), but a subsequent cognate interaction with B cells is required to complete Tfh differentiation (7, 15, 17, 20). This conclusion is based on the demonstration that recognition of immunizing antigen *via* the BCR is required for Tfh formation (20); however, a direct role for B cell antigen presentation has not been shown. To address this question, we made mixed BM chimeras (20% MHC II^−/−^ + 80% μMT BM into irradiated μMT recipient mice) in which the B cell compartment completely lacked expression of MHC class II (B-MHC II^−/−^). To account for the fact that 20% of other lineages in these chimeras also lack MHC class II, we made control MHC II^20%−/−^ chimeras in which 20% of all lineages (including B cells) lacked MHC class II expression (as described in Section “Material and Methods”), as well as wild-type (B-WT) control chimeras. After reconstitution (8–10 weeks), we immunized chimeric mice in order to compare the Tfh response elicited by two antigens, SRBC and ovalbumin peptide-pulsed DC, at day 7 postimmunization. We found that the PD1^hi^ Tfh population failed to develop in the B-MHC II^−/−^ chimeras with both immunization protocols (Figures 2A,B). In contrast, the development of PD1^lo^ Tfh-like population was only slightly impaired in the B-MHC II^−/−^ chimeras compared to WT control chimeras (Figures 2A,B). This tallies with the observation that the migration of CD4 T cells in the follicles of B-MHC II^−/−^ chimeras was unaffected (Figure 2E), as documented previously (32). The lack of B cell antigen presentation and the subsequent loss of the PD1^hi^ Tfh population led directly to an inability of these mice to mount a GC reaction. No GC B cells (as detected by co-expression of GL-7 and the GC-specific transcription factor, Bcl-6) were found in the spleens of B-MHC II^−/−^ chimeras (Figures 2C,D) and histologically no GCs were observed in the spleen (data not shown). Thus, while the generation of PD1^hi^ Tfh requires cognate interactions with B cells, a large proportion of the PD1^lo^ Tfh-like population is generated independently of interactions involving B cell MHC class II.

### PD1^lo^ Tfh-Like Cells Become PD1^hi^ Tfh upon Interaction with MHC Class II^+^ B Cells

**Figure 3:**
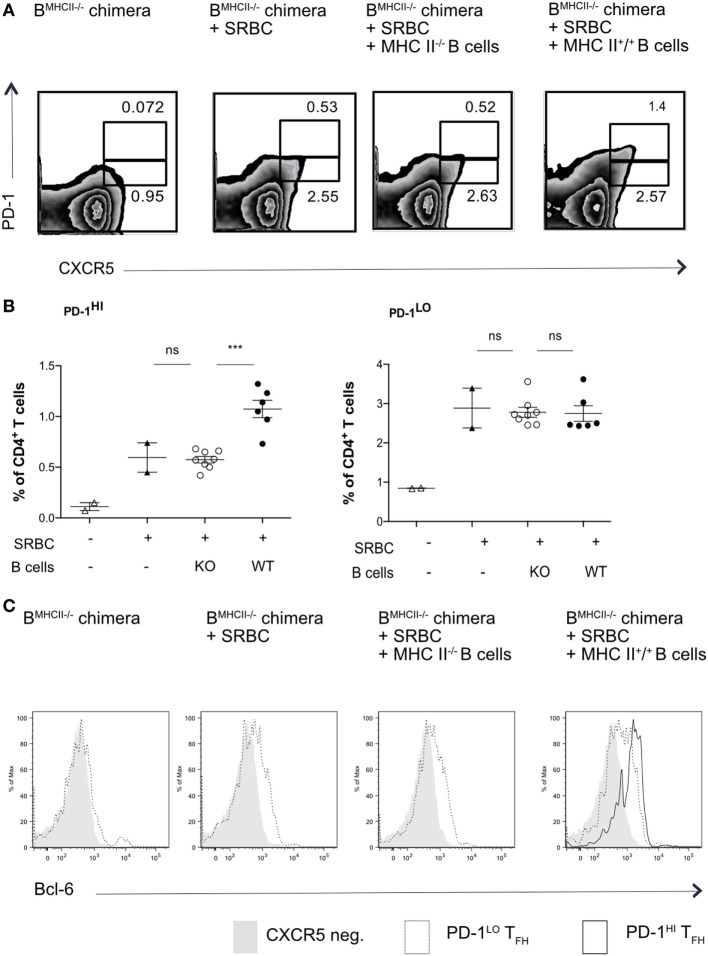
**Transfer of WT B cells rescues PD-1^hi^ T follicular helper cells (Tfh) formation**. B-MCHII^−/−^ chimeric mice were generated as described in the Section “Materials and Methods.” After reconstitution (8–10 weeks), mice were immunized with SRBC and thereafter sorted (97% pure) B cells (1 × 10^6^/mouse i.v.) derived from mice sufficient or deficient of MHC-II were adoptively transferred on the day of SRBC immunization. After 5 days, mice were analyzed for the presence of Tfh-phenotype cells, as described by the co-expression of CXCR5 and PD1 **(A,B)** and the transcription factor, Bcl-6 **(C)**. **(A)** Representative flow cytometry of PD-1^hi^ and PD-1^lo^ Tfh-like cell populations from each of the experimental groups (labeled on top row). **(B)** Summary of PD-1^hi^ and PD-1^lo^ Tfh-like frequencies in each of the experimental groups. **(C)** Representative flow cytometry plots of Bcl-6 expression in PD-1^hi^ (bold black line) and PD-1^lo^ (black line) Tfh and CXCR5^−^ T (gray histogram) cells. The data are pooled from two independent experiments with two animals per group (B-MHCII^−/−^ chimera, B-MHC II^−/−^ chimera + SRBC) or four animals per group (B-MHC II^−/−^ chimera + WT or KO B cells); each dot represents one mouse. Statistical significance was determined by one-way ANOVA with post-ANOVA Tukey’s test for multiple comparisons.

To ask whether PD1^lo^ Tfh-like cells could differentiate into PD1^hi^ cells, we again used MHC II mixed-BM chimeras, in which the B cell compartment lacked MHC II. In the first set of experiments, we adoptively transferred reconstituted MHC II-sufficient (MHC II^+^) or -deficient B cells in reconstituted B-MHC II^−/−^ chimeras just prior to immunization with SRBC. Figures 3A,B show that transfer of a relatively small cohort of MHC II^+^ B cells could drive reconstitution of the PD1^hi^ Tfh compartment after SRBC immunization. The transfer of MHC II^+^ B cells into B-MHC II^−/−^ chimeras had no augmenting effect on the PD1^lo^ Tfh-like population (Figure 3B, right panel) and, as expected, transferring MHC II-deficient B cells had no effect on PD1^hi^ Tfh numbers. Interestingly, although all of the immunized chimeras exhibited Tfh cells expressing intermediate levels of Bcl-6, only those receiving MHC II^+^ B cells showed a Bcl-6^hi^ Tfh population (Figure 3C).

**Figure 4:**
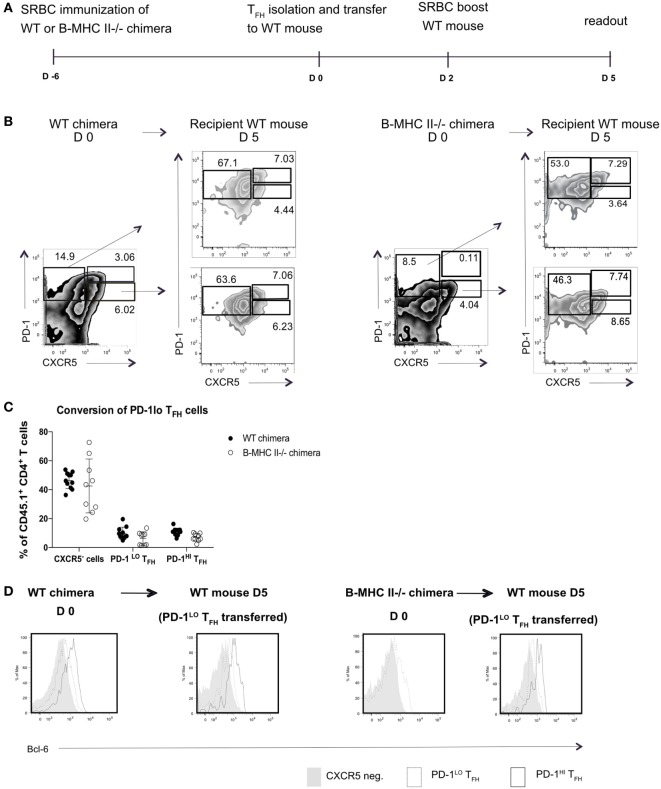
**PD-1^lo^ T follicular helper cells (Tfh) can convert to PD-1^hi^ Tfh and upregulate Bcl6 expression**. **(A)** Diagram of experimental set up for Tfh adoptive transfer. CD45.1^+^ WT or B MHC II^−/−^ chimeras were immunized with SRBC. Six days later, Tfh populations were sorted and transferred to CD45.2^+^ WT recipients. Forty-eight hours post transfer WT recipients were boosted with SRBC, and final analysis was carried out 3 days later. **(B)** Flow cytometry plots of T follicular helper (Tfh) and CXCR5^−^ T cell populations isolated from WT chimera or B-MHCII^−/−^ chimera 6 postimmunization with SRBC (day 0) and after the transfer to congenic WT host and challenge with SRBC (day 5). Transferred PD-1^lo^ Tfh-like population is shown in the lower plot and control CXCR5^−^ group in upper plot (recipient mice). **(C)** Summary of the phenotype acquired by PD-1^lo^ Tfh-like at day 5 post transfer. **(D)** Bcl-6-expression in PD-1^hi^ (bold black line) and PD-1^lo^ (black line) Tfh and CXCR5^−^ T (gray histogram) cells as gated as in B from WT chimeras or B-MHCII^−/−^ chimeras on day 0 (left) or day 5 post transfer (right). Data are representative of two independent experiments (*n* = 4–5, WT chimera) or three independent experiments (*n* = 3–5, B-MHCII^−/−^ chimeras). Each dot represents a single mouse.

This result did not, however, demonstrate that the PD1^hi^ Tfh were derived from PD1^lo^ cells; it was equally possible that the transferred MHC II^+^ B cells elicited *de novo* generation of PD1^hi^ Tfh from naïve T cells. To address this, we next sorted PD1^lo^ Tfh-like cells from either B-WT chimeras or from B-MHC II^−/−^ chimeras (both CD45.1^+^) into CD45.2^+^ WT recipients 6 days postimmunization with SRBC. WT recipients were boosted 2 days after transfer and analyzed 3 days later (Figure 4A). Figure 4B shows that adoptively transferred PD1^lo^ Tfh-like cells isolated from either a WT or B MHC II^−/−^ chimera give rise to significant proportion of PD1^hi^ Tfh (CD45.1^+^) (Figures 4B,C). In this respect, it should be noted that the majority of cells derived from the donor PD1^lo^ Tfh-like cells were characterized as CXCR5-negative activated/effector T cells (Figures 4B,C). As in the previous experiment (Figure 3), the PD1^lo^-derived PD1^hi^ Tfh cells had upregulated the expression of Bcl-6 to levels higher than found in the PD1^lo^ cells (Figure 4D).

### PD1^hi^ but Not PD1^lo^ Tfh-Like Development Depends on Expression of CXCR5 on T Cells

**Figure 5:**
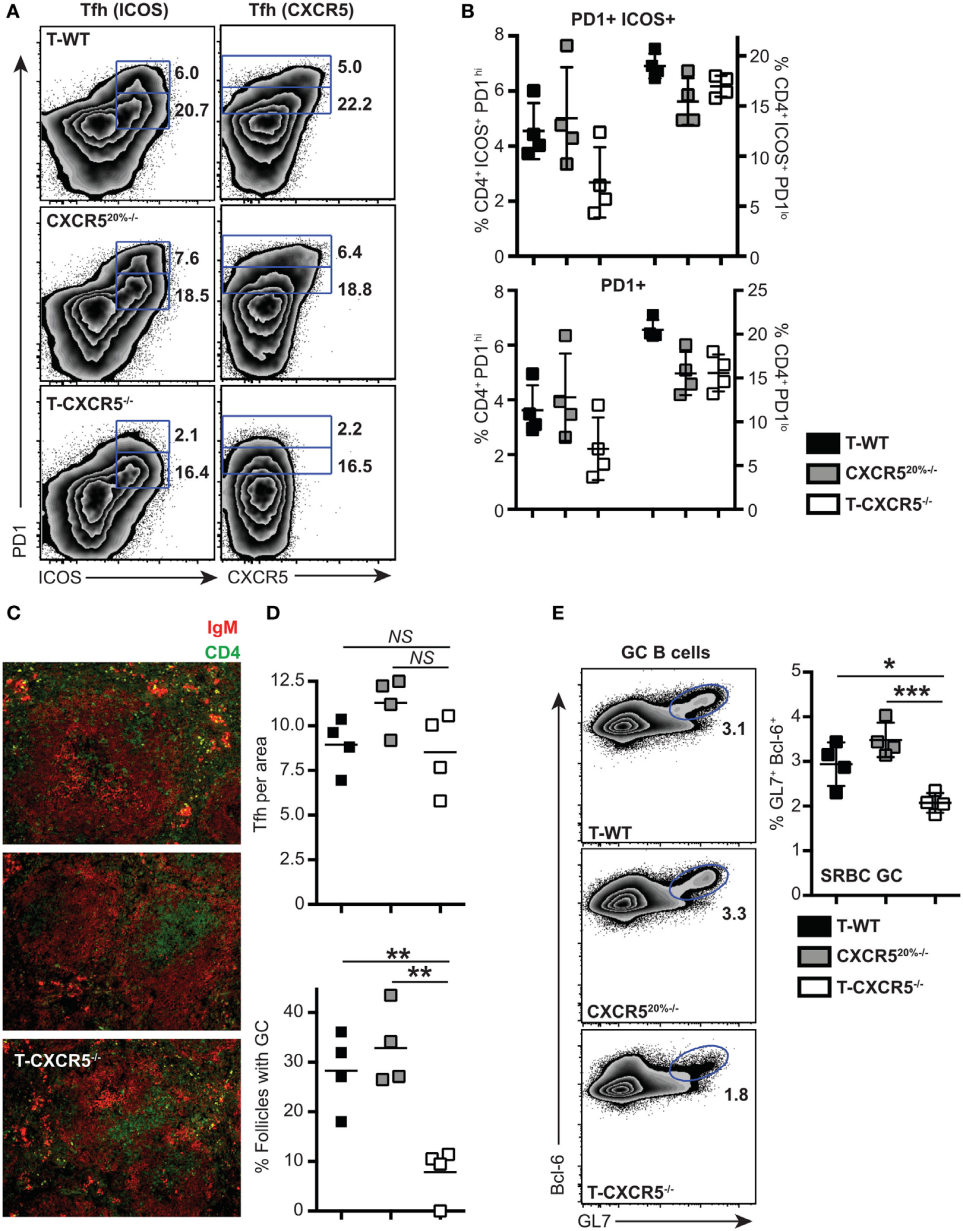
**The influence of CXCR5 expression by T cells on the differentiation of PD1^lo^ and PD1^hi^ T follicular helper cells (Tfh)**. Mixed bone marrow chimeras in which T cells do not express CXCR5 (T-CXCR5^−/−^) or control (T-WT and CXCR5^20%−/−^) were immunized with SRBC. Tfh frequency and phenotype was assessed 7 days postimmunization by flow cytometry for CXCR5, PD1, and ICOS. **(A)** Representative FACS plots showing gating for PD1^hi^ and PD1^lo^ Tfh-like based on co-expression of ICOS (left) and CXCR5 (right). **(B)** Mean frequencies of Tfh among CD4^+^ T cells on groups of four mice. Panel **(C)** shows immunohistochemical staining of splenic sections from SRBC immunized mice for B cell (IgM: red) and T cell (CD4: green) markers. **(D)** Enumeration in histological sections of Tfh per follicle unit area and number of follicles containing a germinal center (GC). **(E)** GCs were also enumerated at day 7 by FACS staining for GL-7 and Bcl-6 of spleen-derived B cells: representative FACS plots and mean frequencies (+scatter) gated on CD19^+^ B cells. Scatter plots **(D,E)** show each point for four separate mice, with the horizontal bar indicating the mean values and error bars SEM. Statistical analysis is by one-way ANOVA where *p < 0.05, **p < 0.01, and ***p < 0.005. Data are representative of two independent experiments.

Few studies have shown that the CXCR5 is not essential for the entry of T cells into follicles (7, 33); instead, interaction with non-cognate B cells *via* ICOS–ICOSL receptor was found required for the T cells to locate to the B cell follicles (33). To understand at what level CXCR5 was important for the differentiation of PD1^hi^ Tfh, we constructed mixed-BM chimeras in which the T cell compartment was completely deficient in CXCR5 (20% CXCR5^−/−^ + 80% CD3ε^−/−^ BM into irradiated CD3ε^−/−^ mice, T-CXCR5^−/−^). In these mice, >80% of the B cells expressed CXCR5 and so B cell follicles formed normally. Control chimeras had a 20% deficit in CXCR5 expression in all cell lineages, including T cells (CXCR5^20%−/−^). In a second control group, T-WT chimera, all the cells of hematopoietic compartment had WT expression of CXCR5. In the T-CXCR5^−/−^ chimeras, Tfh could not be identified on the basis of CXCR5 expression (Figure 5A) and so we stained CD4^+^ T cells additionally for ICOS and PD1. Figure 5A shows that, in the control chimeras, the frequency of cells expressing ICOS and PD1 corresponded well with that expressing CXCR5 and PD1, in both the PD1^hi^ and PD1^lo^ populations. In the T-CXCR5^−/−^ chimeras, the frequency of PD1^hi^ Tfh was reduced (although not to 0), while PD1^lo^ Tfh-like cells remained unchanged in frequency (Figure 5B). In line with the reduced number of PD1^hi^ Tfh, in T-CXCR5^−/−^ chimeras, the number of follicles containing GC was significantly reduced to less than 10% down from over 30% seen in controls (Figures 5C,D). In parallel, GC B cells (as detected by GL7 and Bcl-6 co-expression) were correspondingly reduced in T-CXCR5^−/−^ chimeras compared to control mice (Figure 5E). This is in agreement with previous studies which show that CXCR5 expression is not sufficient for the T cell entry to the follicle, while it is required for optimal B cell responses (7, 34, 35). Thus, PD1^hi^ Tfh responses were observably decreased in the absence of CXCR5, while the generation of PD1^lo^ Tfh-like cells was seemingly unaffected.

### PD1^hi^ GC–Tfh Cannot Give Rise to PD1^lo^ Tfh Like

**Figure 6:**
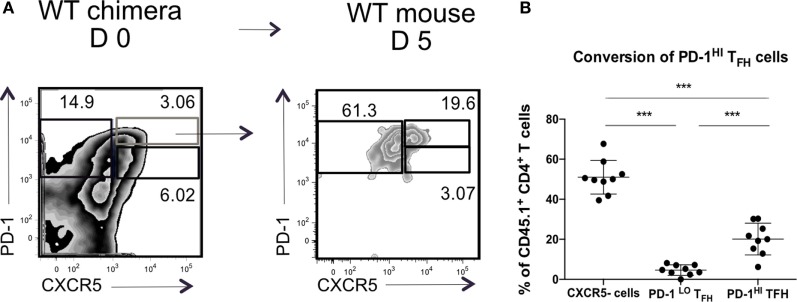
**PD-1^hi^ T follicular helper cells (Tfh) are restricted in their ability to convert to PD-1^lo^ Tfh**. Chimeric mice were generated and treated as described in Figure 4. **(A)** Flow cytometry plots of T follicular helper cells (Tfh) populations isolated from WT chimera on day 0 (left) or day 5 post transfer (right). Transferred PD-1^hi^ Tfh population is showed in blue. **(B)** Summary of the phenotype acquired by PD-1^hi^ Tfh at day 5 post transfer. Data pooled from two independent experiments with four and five animals per group, each dot represents one mouse. Statistical significance was determined by one-way ANOVA with post-ANOVA Tukey’s test for multiple comparisons.

We wondered if the transition through the PD1^hi^ Tfh population was transient, after which the cells reverted to a PD1^lo^ phenotype. To test this, we transferred sorted PD1^hi^ Tfh raised in CD45.1^+^ WT chimeras after SRBC immunization into CD45.2^+^ congenic recipient mice, boosted the recipients with SRBC 48 post transfer, and assessed donor T cell subsets 3 days later (as presented in Figure 4A). After transplantation in CD45.2^+^ WT hosts, the CD45.1^+^ PD1^hi^ Tfh donor cells (or their progeny) gave rise to two populations: PD1^hi^ Tfh and CXCR5-negative activated/effector T cells (Figure 6). Very few donor cells were seen in the PD1^lo^ Tfh-like subset. We, therefore, concluded that the differentiation of PD1^lo^ Tfh-like toward PD1^hi^ GC–Tfh cells was mostly unidirectional and not reversible.

### PD1^lo^ Tfh Like Are Early Effector Cells in Response to *Salmonella*

**Figure 7:**
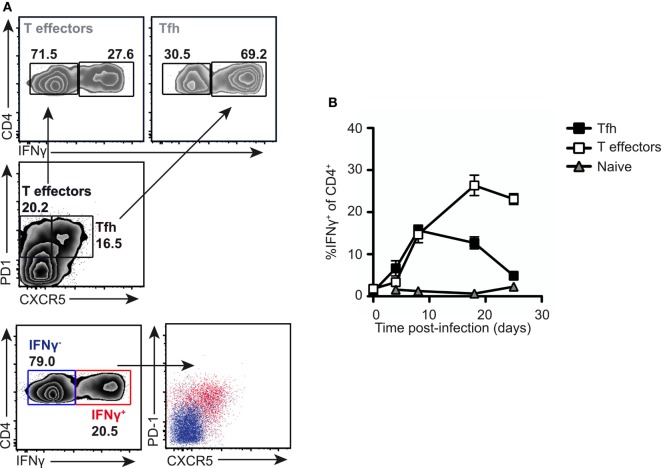
**PD1^lo^ T follicular helper cells (Tfh)-like function as effector cells early in response to *Salmonella***. Analysis of intracellular IFN-γ production in normal mice infected with SL3261. **(A)** Intracellular staining for IFNγ was performed following PMA and ionomycin stimulation on splenocytes from SL3261-infected animals at day 7. Cells were gated on Tfh (PD1^+^CXCR5^+^) versus T effectors (PD1^+^CXCR5^−^) and IFN-γ-producing cells among these populations enumerated (top panel). Overlays (lower panel) of IFN-γ producers (red) versus non-producers (blue) revealed that the majority of cytokine positive cells were PD1^+^CXCR5^+^. **(B)** Kinetics of IFN-γ production in splenic CD4 T cells were analyzed. Cells were distinguished by CXCR5 and PD1 expression as Tfh (CXCR5^+^ PD-1^+^, black squares), T effectors (CXCR5^−^ PD-1^+^, white squares), and naive T cells (CXCR5^−^ PD1^−^, gray triangles). Data points (**B**) represent mean of *n* = 5 with error bars SEM. Presented data are representative of three independent experiments.

To gain insight into the function of the PD1^lo^ Tfh-like population, we returned to the *Salmonella* infection where the Tfh response stalls in the PD1^lo^ stage. The predominant response to *Salmonella* is IFN-γ and IFN-γ-producing effector cells start playing crucial role in elimination of pathogen 2 weeks postimmunization with *Salmonella* (9, 36). Therefore, we assessed the production of IFN-γ in T cell subsets following infection. As can be seen in Figure 7A, the PD1^lo^ Tfh-like cells made up the largest population of IFN-γ-producing T cells at day 7 postinfection (69% of IFN-γ-producers, compared to 27% of the non-Tfh cells, in the example shown). Conversely, most of the IFN-γ-producing T cells were PD1^lo^ CXCR5^+^ Tfh cells at this time point (Figure 7A, lowest two panels). However, this changed over time postinfection (Figure 7B). Beyond day 7, the proportion of PD1^lo^ Tfh-like cells making IFN-γ declined, while non-Tfh effector cells increased IFN-γ secretion totally dominating the response by day 20. Thus, a rapid, early but transient cytokine response is made by the PD1^lo^ Tfh-like cells. This is reminiscent of the “memory precursor” population of CD8 effector T cells defined by Kaech and colleagues (37).

### Transcriptional Profiling of PD1^hi^ and PD1^lo^ Tfh-Like Subsets

**Figure 8:**
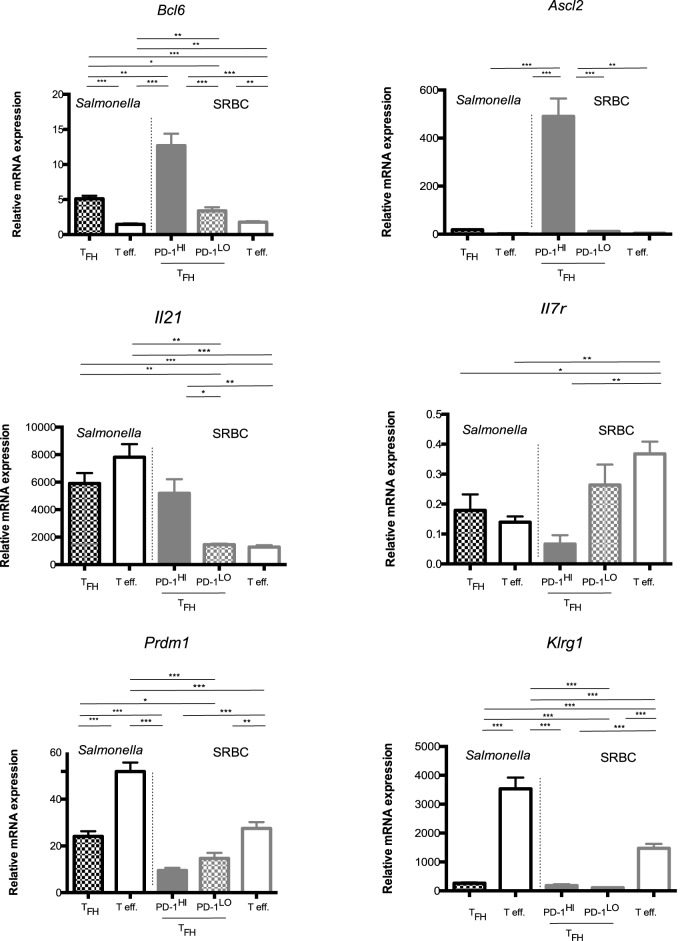

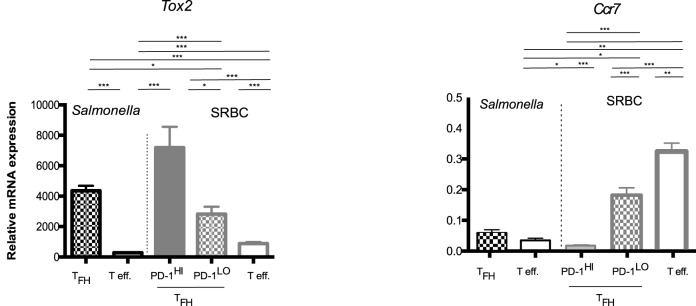
**Memory potential of PD1^hi^ and PD1^lo^ T follicular helper cells (Tfh) like**. Quantitative RT-PCR analysis of mRNA for *Bcl6, Ascl2, Il21, Il7R, Prdm1, Klrg1*, and *Tox2* in CD4^+^ T cells sorted (>95% purity) from the pooled spleen of mice 6 days after immunization with SRBC or *Salmonella*. Cells were sorted as naïve T cells (CD62L^+^CD44^−^), effector cells (PD1^+^CXCR5^−^), and Tfh cells (PD1^+^CXCR5^+^), with Tfh being further separated on the basis of PD1 expression into CXCR5^+^PD1^hi^ and CXCR5^+^PD1^lo^ Tfh-like. Results are presented relative to the average expression of the housekeeping genes UBC (ubiquitin C) mRNA and 18S ribosomal RNA. Final mRNA expression values were calculated relative to the transcripts found in naïve T cells (CD62L^hi^ CD44^lo^) isolated from unimmunized mice. Bars represent mean expression with error bars indicating SEM. Statistical analysis is by unpaired Student’s *t*-test, where *p < 0.05, **p < 0.01, ***p < 0.001. Data are representative of two independent experiments with similar results with three to five biological replicates per gene.

To investigate the possibility that the PD1^lo^ Tfh-like population represented a “memory precursor effector,” we performed mRNA expression profiling using microarray on sorted PD1^hi^ and PD1^lo^ Tfh-like populations following SRBC immunization (>98% and >96% pure, respectively) and the PD1^lo^ Tfh-like cells arising during *Salmonella* infection (>95% pure). These subsets were compared to non-Tfh effectors (PD1^+^ CXCR5^−^) in the relevant response (both populations 98% pure). As an additional control, we have sorted naïve CD4^+^ T cells from unimmunized mice (CD4^+^ CD62L^hi^ CD44^lo^, 99% pure). The full data set is available at: https://www.ncbi.nlm.nih.gov/geo/query/acc.cgi?acc=GSE62961. We independently confirmed the microarray data by performing Q-RT-PCR to measure “effector/memory”-associated gene expression in these subsets, as illustrated in Figure 8.

The PD1^hi^ Tfh generated upon SRBC immunization showed a quite distinct profile from all the other “Tfh” subsets. They expressed by far the highest levels of *Bcl6* mRNA and were the only population exhibiting expression of the recently described Tfh-specific transcription factor, *Ascl2* (Figure 8). In the SRBC response, the PD1^hi^ Tfh cells were expressing significantly more IL-21 mRNA than seen in SRBC PD1^lo^ Tfh-like and effector cells; however, this was not the case in the *Salmonella* response where both effectors and PD1^lo^ Tfh-like transcribed this cytokine gene to comparable levels, as detected in SRBC PD1^hi^ Tfh cells (Figure 8). So, although the SRBC PD1^lo^ Tfh-like and *Salmonella* Tfh cells shared some characteristics with PD1^hi^ Tfh, our microarray analysis (Figure S2 in Supplementary Material) strongly suggests that PD1^lo^ and PD1^hi^ Tfh have potential to be functionally distinct. With regard to markers that identify memory versus effector potential, in both responses *Prdm1* (BLIMP1) and *Klrg1* were expressed most highly in the effector cells. Even if PD1^lo^ Tfh-like showed some degree of *Prdm1* upregulation with respect to naïve T cells, levels remained approximately 50% lower than seen in effectors, while *Klrg1* expression remained stably low in PD1^lo^ Tfh-like cells. *Tox2*, previously associated with memory potential, was most highly expressed in PD1^hi^ Tfh (SRBC). However, PD1^lo^ Tfh-like cells also expressed elevated *Tox2* levels compared to effectors. Importantly, while the PD1^hi^ Tfh expressed little message for *Il7r*—indicating little potential to enter the memory pool (37, 38)—PD1^lo^ Tfh-like cells maintained comparatively high levels of *Il7r* mRNA. Thus, *Salmonella* and SRBC PD1^lo^ Tfh-like expressed genes characteristic of both memory (*Tox2, Il7r*) and short-lived effector cells (*Klrg1, Prdm1*), although the latter were expressed at much lower levels than in *bona fide* effector cells. A wider range of genes expressed in the sorted Tfh populations is shown as a microarray heat map in Figure S2 in Supplementary Material. Collectively, our data confirm the distinct nature of the PD1^hi^ Tfh population (the classical GC–Tfh) and emphasize that the PD1^lo^ Tfh-like cells (*Salmonella* and SRBC) express an mRNA signature reminiscent of memory precursors, including differential expression of *Tox2, Slamf6, Id2, Bcl6* (in comparison to naïve T cells) (39), and *Il7r*.

## Discussion

The identification of a subset of T cells (Tfh) that localized to B cell follicles, was dependent on B cells for development and/or survival, and seemed specialized to provide help to B cells has led to the generally accepted idea that T cells entering follicles do so to support B cell responses, in particular the GC reaction (8, 13). Our current study suggests a more complex picture, emphasizing that what we currently call Tfh are a heterogeneous group of cells that may differ in phenotype and function depending on environmental cues and may not have as their primary function that of helping B cells. We first noticed this in the response to *Salmonella* infection where there is a rapid and significant induction of CXCR5^+^ PD1^+^ T cells (Tfh-like cells) concomitant with an appearance of activated T cells in B cell follicles. In infected mice, the Tfh-like responses, although associated with B cell follicles, did not support GC formation. Interestingly, when compared to the Tfh cells generated following immunization with SRBC or Ag-pulsed DC, the *Salmonella* Tfh-like cells fall within a previously described PD1^lo^ population and almost completely lack the PD1^hi^ population that predominates in the Tfh generated by SRBC immunization (9). Like PD1^hi^ cells, the PD1^lo^ Tfh-like population was found in B cell follicles and was critically dependent on the presence of B cells for their persistence (demonstrated by anti-CD20 depletion of B cells). Pre-Tfh found in extrafollicular sites also have a PD1^lo^ phenotype (9). To investigate the developmental and functional relationship of the PD1^lo^ and PD1^hi^ Tfh populations, we used SRBC or Ag-pulsed DC immunizations as these generate both populations.

The molecular basis of the B cell dependence of both populations was investigated further using mice in which Ag-presentation by B cells was precluded (B cells were MHC II-deficient). We found that the PD1^hi^ population required B cell Ag-presentation, while the PD1^lo^ Tfh-like population in general did not. Transfer of even a small number of MHC II^+^ B cells into the B-MHC II^−/−^ chimeras restored the differentiation of PD1^hi^ Tfh cells. It turned out that (after adoptive transfer) both PD1^lo^ Tfh-like (CXCR5^+^) and activated non-Tfh (CXCR5^−^) cells could give rise to the PD1^hi^ Tfh cells. However, while PD1^lo^ Tfh-like can become PD1^hi^ Tfh cells, the differentiation is unidirectional as adoptively transferred PD1^hi^ Tfh did not become PD1^lo^ cells. This may indicate that they are end stage effectors or possibly, if they lose markers, they may become CXCR5^−^ and move out of the follicular T cell compartment.

In trying to prevent T cells from entering B cell follicles, we constructed BM chimeras in which the T cell compartment was deficient in CXCR5 (T-CXCR5^−/−^ chimeras). Initially, to our surprise, we found this did not restrict their localization to follicles. However, we now surmise that this fits with the observation that ICOS ligation by B cells causes changes in cell motility (filopodia formation) that lead to CXCR5-independent migration into the outer regions of the follicle (33). This CXCR5-negative follicular T cell population exhibits a dramatic reduction in the PD1^hi^ subset, while the PD1^lo^ T cell population is unaffected, and, as a consequence, there is impaired support for GC formation (Figure 4). This suggests that, although CXCR5 expression is dispensable for follicular entry, it is needed for the final stage of Tfh differentiation into the PD1^hi^ GC–Tfh population or alternatively, for the localization of these cells to the follicular center next to FDC or GC B cell precursors. Interestingly, CCR7 expression, which is low but detectable in the PD1^lo^ population, is extinguished completely in PD1^hi^ Tfh cells (Figure 8; Figure S2 in Supplementary Material). This is in keeping with previous studies showing that CXCR5 is overexpression in naïve T cells is not sufficient for the follicular entry, which occurs only with CCR7 downregulation (7). Taken together with the data from our B-MHC II^−/−^ chimeras and studies from other labs (8), we propose a multistage process of Tfh development in which, following Ag-specific activation by DCs, non-cognate, ICOS-L-dependent interactions with B cells (33) give rise to PD1^lo^ Tfh-like cells that inhabit the mantle zone of follicles. Upon subsequent cognate interaction with B cells, some of these cells become GC-supporting PD1^hi^ Tfh cells. While the maintenance of CD4 T cells within the B cell follicle seems to be ICOS dependent as a result of downregulation of the transcription factor, Krüppel-like factor 2 (KLF2) (40, 41), the final transition to PD1^hi^ Tfh cells and their positioning in the GC requires a TCR signal delivered by B cell MHC II/peptide. Whether these TCR signals cause further downregulation of KLF2 expression and activity or whether the transient downregulation of FOXO1 signals (42) are involved in this final differentiation step requires further investigation.

Our previous work highlights the importance of cognate interactions with B cells for robust T cell memory (18, 19, 22). The demonstration that PD1^lo^ Tfh-like cells and PD1^hi^ Tfh are similarly dependent on B cells and their MHC II expression raises in our minds the possibility that either or both of these Tfh populations have potential as memory precursors. The transcriptional analysis we have done especially suggests that the PD1^lo^ (more than PD1^hi^) populations bears most resemblance to memory precursors. First, PD1^lo^ Tfh-like population preserves the potential to produce effector cytokines (IFN-γ). Second, PD1^lo^ Tfh-like cells express wide range of markers found in memory cell pool given relatively high expression of *Id2*, IL-7R, and IL-2Rα (37). Third, the PD1^lo^ Tfh-like cells do not upregulate terminal differentiation transcription factor *Blimp1* and exhaustion marker Klrg1. Finally, they are able to reconstitute both PD-1^hi^ Tfh and CXCR5^−^ effector T cell population while preserving the self-renewal potential. Resemblance of PD1^lo^ Tfh to memory cells is further supported by the observation (43) that CXCR5 expression on CD4 T cells was associated with the ability to provide enhanced support for B cell recall responses. More directly Jenkins and colleagues (28, 44) have shown that CXCR5^+^CCR7^+^Tbet^lo^PD1^lo^ T cells were precursors of central memory cells (Tcm) and went further to demonstrate that the generation of CXCR5^+^ Tcm was B cell-, ICOS-, and Bcl-6-dependent (28). They suggested that these cells were not in follicles, in contrast to the major PD1^lo^ Tfh-like subset highlighted here. It was also clear from this study that the PD1^hi^ Tfh population did not give rise to central memory T cells. A recent study by Choi et al. (39) also concludes that Tfh cells are major contributors to the CD4 memory T cell pool during an antiviral response. Their memory precursor cells expressed high levels of Bcl-6 and also IL-7Rα. Further evidence for the memory within Tfh lineage and Tfh persistence in the absence of antigen are provided by transfer experiments with IL-21-GFP reporter mice (45), Bcl-6 YFP OT.II reporter mice (46), and OT.II transgenic mice (41). We proposed based on our data that memory precursors are found in the PD1^lo^ Tfh-like-like subset rather than the PD1^hi^ Tfh as the latter express very low levels of IL-7Rα (Figure 8; Figure S2 in Supplementary Material). However, the role of IL-7R in memory specification in the CD4^+^ T cell compartment is controversial, as a study by Marshall et al. indicated that IL-7R low and high cells converted equally well into memory cells during a viral infection (47). Finally, we found that PD1^hi^ Tfh cells expressed the highest levels of IL-4 mRNA (alongside IL-21 mRNA), suggesting a more differentiated phenotype than PD1^lo^ Tfh-like cells, which is also in agreement with studies published by Haynes et al. (7).

The different nature of Tfh raised in *Salmonella* model and SRBC immunization is an intriguing issue. The study by Ryg-Cornejo and colleagues shows that in severe malaria infection Tfh cells are stalled at the precursor stage due to high level of pro-inflammatory cytokines (IFN-γ and TNF-α) (48). Importantly, this causes reduction in GC and antibody production. These pre-Tfh cells are also PD-1^lo^ and CXCR5-intermediate, express Bcl6 and IL-21 (48). Since *Salmonella* infection is also a Th1-driven model, this could explain the lack of PD-1^hi^ Tfh cells and, as a consequence, impairment in the GC formation. Another aspect of *Salmonella* infection is splenomegaly associated with the disruption of splenic architecture. However, we think it is unlikely to be the reason for the lack of PD-1^hi^ Tfh, as immunization with lower doses of *Salmonella* or heat-killed *Salmonella* (which do not result in splenic disruption) neither elicit a PD-1^hi^ Tfh population nor GC formation.

Given that T cell memory is largely B cell-dependent and that the majority of B cells are found within the lymphoid follicles, it should come as no surprise that this site is also the crucible of central T cell memory formation. That B cells form ectopic follicular structures at inflamed sites in the periphery raises the tantalizing possibility that they may play a similar role in the generation and maintenance tissue-resident memory cells (49). In conclusion, our data delineate a step-wise differentiation of the T cells along the Tfh-like pathway. This differentiation program leads to heterogeneity in phenotype and function of the Tfh cells with implications for T cells beyond “helper” function.

## Materials and Methods

### Mice and Mixed BM Chimeras

C57Bl/6, CD45.1, μMT, CD3ε^−/−^, CXCR5^−/−^, and MHC II^−/−^ mice were bred and maintained at the School of Biological Sciences Animal Facility, University of Edinburgh (Edinburgh, UK). Mice were aged 6–10 weeks at the start of procedures. Experiments were covered by a Project License granted by the UK Home Office under the Animals (Scientific Procedures) Act of 1986 and approved locally by the University of Edinburgh Ethical Review Committee. Mice with a B cell-specific MHC II or T cell-specific CXCR5 deficiency were generated using the mixed-BM chimera system as described previously (18, 50). In brief, irradiated μMT or CD3ε^−/−^ recipients were reconstituted with a mixed inoculum of BM (20% MHC II^−/−^ or 20% CXCR5^−/−^ BM and 80% μMT or CD3ε^−/−^ BM, respectively). In a second control group, B-WT and T-WT chimera, all the cells of hematopoietic compartment had WT expression of MHC II or CXCR5 (20% WT + 80% μMT or 80% CD3ε^−/−^ BM in a μMT or CD3ε^−/−^ host, respectively). To control for a partial deficiency in the non-B/T cell compartment, a third control group with a 20% deficiency in all hematopoietic cells was made (designated MHC-II^20%−/−^ and CXCR5^20%−/−^; 20% MHCII^−/−^; or CXCR5^−/−^ BM + 80% WT BM in a μMT or CD3ε^−/−^ host, respectively). Chimerism was confirmed by flow cytometric analysis of B cell MHC-II expression or T cell CXCR5 expression.

### Immunizations

The aroA attenuated strain of *Salmonella* enterica serovar Typhimurium (SL3261) was used for all infections (18, 51). Bacteria were grown as stationary-phase 16-h cultures in Luria-Bertani (LB) broth (Difco Laboratories, Surrey, UK). Animals were injected intravenously (i.v.) with ~1 × 10^6^ colony-forming units, diluted in PBS. Infectious dose was determined by plating bacteria onto LB agar plates and culturing overnight at 37°C. Experiments involving *Salmonella* were carried out according to Biosafety Level 2 requirements. Ag-pulsed DC immunizations were carried out using BM-derived dendritic cells, generated by a modified version of the procedure developed by Inaba et al. (52). Briefly, BM was harvested from femurs and tibia of C57Bl/6 mice. Approximately, 2 × 10^6^ cells were cultured in 10 ml RPMI, further supplemented with 10% FCS and 20 ng/ml granulocyte macrophage colony-stimulating factor (GM-CSF, Peprotech EC Ltd., London, UK). Plates were incubated at 37°C in humidified 5% CO_2_ atmosphere. Medium was replaced with fresh GM-CSF containing medium on days 3, 6, and 8. On day 10, DC were harvested and cultured at 2 × 10^6^ cells/ml with OVA peptide (323–339) at 10 µg/ml and LPS at 1 µg/ml for 16 h. The following day, cells were harvested and extensively washed prior to transfer. Approximately, 2 × 10^6^ cells were injected i.v. in a total volume of 200 µl. SRBC immunizations were carried out as previously described (53). Briefly, SRBC were washed twice with PBS prior to immunization with 1 × 10^9^ SRBC per mouse *via* the intraperitoneal route.

### B Cell Depletion

B cells were depleted by single i.v. injection of 250 µg anti-mouse CD20 (clone 18B12) or isotype control (clone 2B8, both from Dr. Marylin Kehry, Biogen IDEC Inc.) on day 2, day 6, or day 10 of infection. Depletion was confirmed by quantifying peripheral blood B cells by flow cytometry based on CD19 and B220 expression.

### Flow Cytometric Staining

Before staining, cells were washed in ice-cold PBS and stained with Live/Dead fixable aqua fluorescent stain (Invitrogen, Carlsbad, CA, USA) for 20 min at 4°C. After washing in FACS buffer (PBS with 0.05% sodium azide and 3% FCS), cells were stained with anti-CXCR5 biotin, anti-GL7 FITC, anti-ICOS PE, anti-PD1 PE-Cy7, anti-CD4 APC-Cy7, and CD19-pacific blue (BD Biosciences) in the presence of Fc receptor blocking antibody (clone 2.4G2, in-house) for a further hour at 4°C, then washed. Streptavidin-APC was added, and samples incubated for 30 mins on ice. Staining for intracellular transcription factors and/or cytokines (Bcl-6 and IFN-γ) were performed following surface staining using fix/perm buffer set (eBioscience, San Diego, CA, USA). Samples were acquired on a LSR II flow cytometer (BD Bioscience, San Diego, CA, USA) using BD FACS-Diva software, and data analyzed using FlowJo software (Tree Star Inc., San Carlos, CA, USA).

### Cell Isolation, T and B Cell Sorting, and Adoptive Transfer

Single cell suspensions were prepared from the spleen by manual disruption in IMDM plus 2% FCS and penicillin/streptomycin. For isolation of individual Tfh populations, flow cytometry associated sorting was used. Tfh populations were isolated at day 6 post SRBC immunization from WT C57Bl/6 mice, MHC II-deficient mice (MHC II^−/−^), or mixed-BM chimeras. The CD4 T cell fraction was enriched by Dynabeads (Invitrogen, Carlsbad, CA, USA) depletion of other cell populations prior to sorting; splenocytes (1 × 10^8^/ml) were labeled with anti-MHCII (clone M5114) and anti-CD8 antibodies (clone 53.6.72) prior to incubation and separation with anti-rat IgG Dynabeads (bead:cell ratio 1:2). This procedure enriched T cells to around 65%. Thereafter enriched T cells were stained using monoclonal antibodies directed against CD4, CXCR5, and PD1 necessary to identify Tfh subsets which were then sorted on a FACS ARIA II (BD Biosciences) as indicated in Figure 4. Specific gates were used to further distinguish and sort the PD1^hi^ and PD1^lo^ populations in the SRBC response as indicated. In adoptive transfer experiments (Figures 4 and 6), 0.4 × 10^6^ sorted T cells (purity >95%) per mouse were injected i.v. in 200 µl of sterile PBS (Sigma-Aldrich, St. Louis, MO, USA). For Q-RT-PCR and microarray analysis, the enriched CD4 T cell populations were FACS-sorted as above. Tfh cells sorted for RNA isolation were stored as cell pellets or in TRIzol reagent (Invitrogen) at −80°C until extraction process. B cells were isolated by magnetic separation (positive selection) with anti-CD19 MicroBeads (Miltenyi Biotec) as per manufacturer’s instruction. Isolated B cells were over 97% pure.

### Immunohistology and Quantification

Spleens were placed in cryo-molds (VWR, Lutterworth, UK) in OCT-embedding medium (VWR) and stored at −80°C until required. Tissue sections of 5 µm in thickness were cut, dried, and fixed in acetone, before being stored at −20°C until use. Sections were blocked in 1% BSA for 15 min and then stained with antibodies diluted in 1% BSA. Between staining with primary and secondary antibodies, and after secondary antibody staining, slides were washed 3 times for 5 min each by immersion in PBS. After staining, cover slips were mounted using Mowiol (Hoechst, Frankfurt, Germany) containing 2.5% DABCO (Sigma-Aldrich, St. Louis, MO, USA). Slides were viewed on an Olympus BX50 microscope under reflected light fluorescence, and images captured using OpenLab software (Improvision, Walthman, MA, USA). Tfh cells were analyzed using sections stained with anti-IgM Texas-Red (Southern Biotechnology Associates, Birmingham, AL, USA) and anti-CD4 biotin (Biolegend) for 1 h followed by Streptavidin FITC (Southern Biotechnology Associates, Birmingham, AL, USA) for 30 min. Follicular size was measured using OpenLab software, and CD4^+^ cells residing within the follicles were counted. Quantification of GC was performed by staining with anti-IgM Texas-Red, anti-CD4 biotin, and PNA FITC (Vector Laboratories Inc., Burlingame, CA, USA) before Streptavidin AlexaFluor 350 (Invitrogen, Carlsbad, CA, USA) was added. Sections were measured and the number of GC per square millimeter was calculated.

### Quantitative Real-time PCR and Microarray Analysis

Total RNA was extracted from sorted T cells stored as a cell pellet or in TriZol (Invitrogen) using RNeasy mini kit (Qiagen). For the microarray analysis, for each subset of interest, cells were sorted in three independent biological repeats. The quality of any recovered RNA was assessed using the Bioanalyser 2100 on a RNA-Nano chip (Agilent technologies), and only preparations with RNA integrity number >8 were considered acceptable for further processing. Extracted RNA was converted to cDNA and hybridized to Affymetrix Mouse Gene ST 1.1 array at ARK Genomics Technologies (The Roslin Institute, University of Edinburgh) according to manufacturer’s protocol. Arrays were scanned with a GeneChip Scanner 3000 (Affymetrix) according to manufacturer’s protocol. Data were processed using rma and limma packages in R/Bioconductor. Pairwise group comparisons were undertaken using linear modeling. Subsequently, empirical Bayesian analysis was applied, including vertical (within a given comparison) *p* value adjustment for multiple testing, which controls for false discovery rate, using the limma Bioconductor package. The normalized data are log2 values for the probes reliably detected on all arrays, processed using rma followed by quantile normalization.

In Q-RT-PCR experiments, from extracted RNA, cDNA was synthesized using Superscript First Strand Synthesis System (Invitrogen, Thermo Fisher Scientific). Gene expression was examined with TaqMan assays (Applied Biosystems, Thermo Fisher Scientific), and Q-PCRs were run on a LightCycler machine (Roche). Ribosomal RNA, 18S and ubiqutin-C mRNA from GeNorm kit (Primer Design) were used as reference genes to normalize mRNA levels. Differences between the experimental samples were analyzed using delta delta Ct method (54) and final mRNA expression values were calculated relative to the transcripts found in naïve T cells (CD62L^hi^ CD44^lo^) isolated from unimmunized mice. PrimeTime Q-PCR assays (IDT) were used for the gene detection (full list of assays is available in Table S1 in Supplementary Material).

### Statistics

The Student’s unpaired *t*-test and two-way ANOVA tests were used to calculate significance values where appropriate. Throughout, *p*-values are illustrated as follows: *p = 0.01 to 0.05, **p = 0.001 to 0.01, ***p < 0.001, NS, not significant (*p* > 0.05).

## Author Contributions

MT, TB, VM, SB, SC, and JR performed the experiments. MT, TB, VM, SC, AI, and DG analyzed the data and interpreted the results. MT, TB, and AI prepared the figures. DG designed the research. DG, TB, and MT wrote the manuscript.

## Conflict of Interest Statement

The authors declare that the research was conducted in the absence of any commercial or financial relationships that could be construed as a potential conflict of interest.

## Supplementary Material

Figure S1 **T follicular helper cell (Tfh) development during the response to *Salmonella* is not associated with help for B cells**. C57Bl/6 mice were infected with SL3261 and the Tfh and B cell responses measured over the subsequent 11 weeks. **(A)** Phenotype and frequency of Tfh in *Salmonella* and SRBC immunized mice at day 7 was determined by flow cytometery for PD1 and CXCR5 and histological analysis of splenic follicles stained with anti-IgM (red) and anti-CD4 (green). Values represent frequency of PD1^hi^ and PD1^lo^ among CD4 cells, respectively. **(B)** Tfh cell frequencies in the spleens were quantified by flow cytometery (upper panel) and histology (lower panel). B cell responses were quantified by measuring *Salmonella*-specific IgG by ELISA (upper panel) and enumerating germinal center (GC) in spleen sections by histology (lower) panel. **(A,B)** Data points represent mean of *n* = 5 with error bars SEM. Presented data are representative of three independent experiments.

Figure S2 **Transcriptional profile analysis of T follicular helper cell (Tfh) subsets by Affymetrix microarray**. T cell subsets were isolated by FACS as described in the online methods, and extracted RNA was subjected to Affymetrix microarrays (Mouse Gene 1.1 ST). Data were processed for quality control with arrayQualityMetrics package in Bioconductor and subsequent normalization was achieved using robust multi-array average expression measure. Samples showing similar profiles across analyzed genes are placed next to each other. Heat map represents expression of the transcripts relevant for effector/memory T cell generation. Colors show sample intensities with blue being the lowest (downregulated genes) and red the highest (strongly upregulated genes). Yellow and orange progressively signal intermediate levels of gene upregulation.
