# Supplementary material for: Heterogeneity of Phenotype and Function Reflects the Multistage Development of T Follicular Helper Cells
Source: Front Immunol. 2017 Apr 28;8:489. doi: 10.3389/fimmu.2017.00489 (PMC5408024; doi:10.3389/fimmu.2017.00489)
Supplement: Supplementary file 1 [file Table_1.DOCX]

Supplementary Table 1. qPCR primer sequences (Integrated DNA Technologies, Iowa, USA).

| Gene | Forward primer 5’-3’ | Reverse primer 5’-3’ |
| --- | --- | --- |
| *Bcl6* | AGTCACATTCGTTGCAGAAGA | CAGAGATGTGCCTCCATACTG |
| *Tox2* | GATTGGGAGGCGTTATAGGAG | GAAGTTTGATGGTGACAGTGC |

Supplementary Table 2. PrimeTime® qPCR assay numbers (Integrated DNA Technologies, Iowa, USA)

| Gene name | PrimeTime qPCR assay number |
| --- | --- |
| *Ascl2* | Mm.PT.58.11676006.gs |
| *Ccr7* | Mm.PT.58.31257202 |
| *Il7r* | Mm.PT.56a.14297778 |
| *Il21* | Mm.PT.56a.7853071 |
| *Klrg1* | Mm.PT.56a.30803964 |
| *Prdm1* | Mm.PT.56a.10253822 |
