# Supplementary figures and images for: Heterogeneity of Phenotype and Function Reflects the Multistage Development of T Follicular Helper Cells
Source: Front Immunol. 2017 Apr 28;8:489. doi: 10.3389/fimmu.2017.00489 (PMC5408024; doi:10.3389/fimmu.2017.00489)

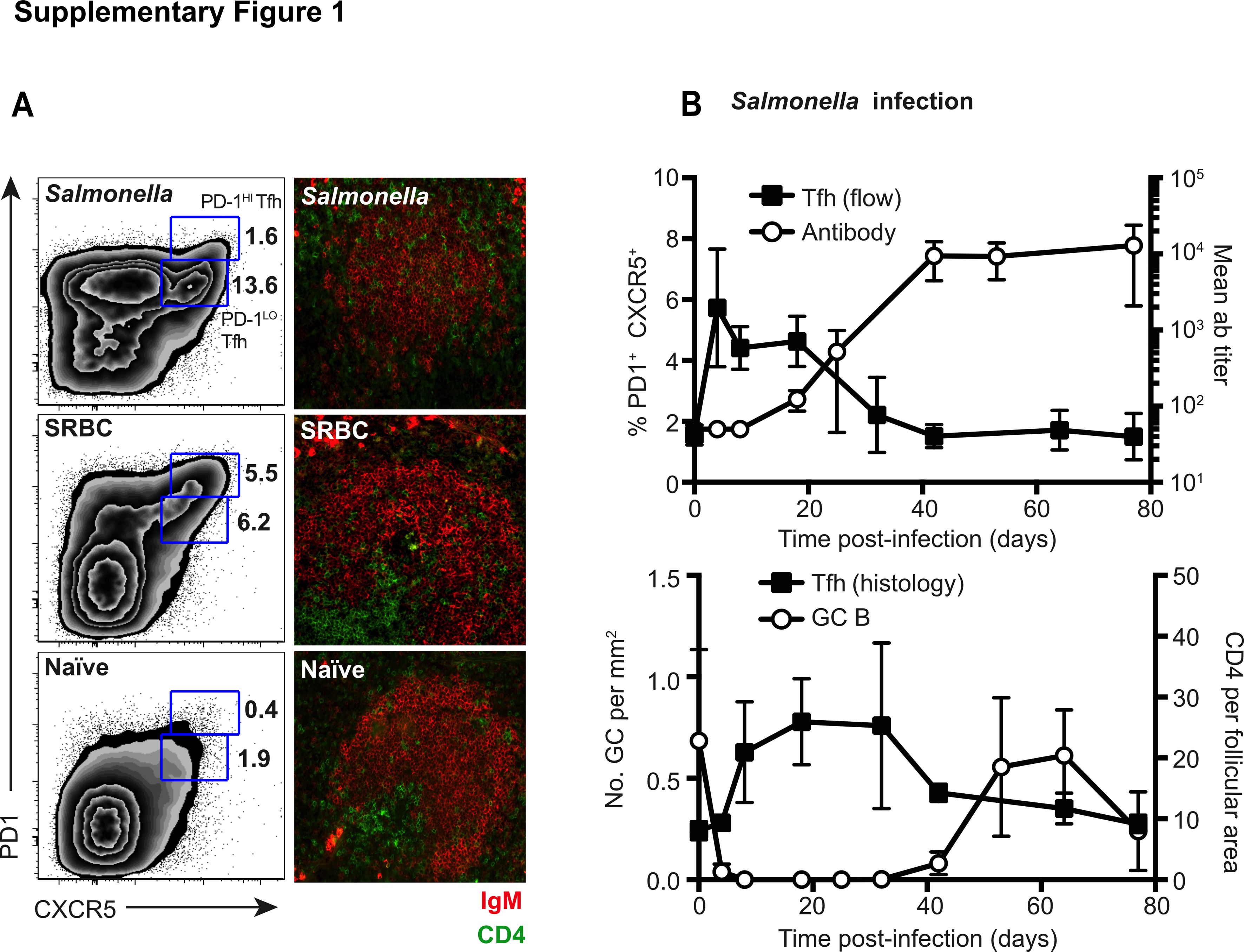

Supplement: Figure S1 — T follicular helper cell (Tfh) development during the response to Salmonella is not associated with help for B cells. C57Bl/6 mice were infected with SL3261 and the Tfh and B cell responses measured over the subsequent 11 weeks. (A) Phenotype and frequency of Tfh in Salmonella and SRBC immunized mice at day 7 was determined by flow cytometery for PD1 and CXCR5 and histological analysis of splenic follicles stained with anti-IgM (red) and anti-CD4 (green). Values represent frequency of PD1hi and PD1lo among CD4 cells, respectively. (B) Tfh cell frequencies in the spleens were quantified by flow cytometery (upper panel) and histology (lower panel). B cell responses were quantified by measuring Salmonella-specific IgG by ELISA (upper panel) and enumerating germinal center (GC) in spleen sections by histology (lower) panel. (A,B) Data points represent mean of n = 5 with error bars SEM. Presented data are representative of three independent experiments. [file Image_1.TIFF]

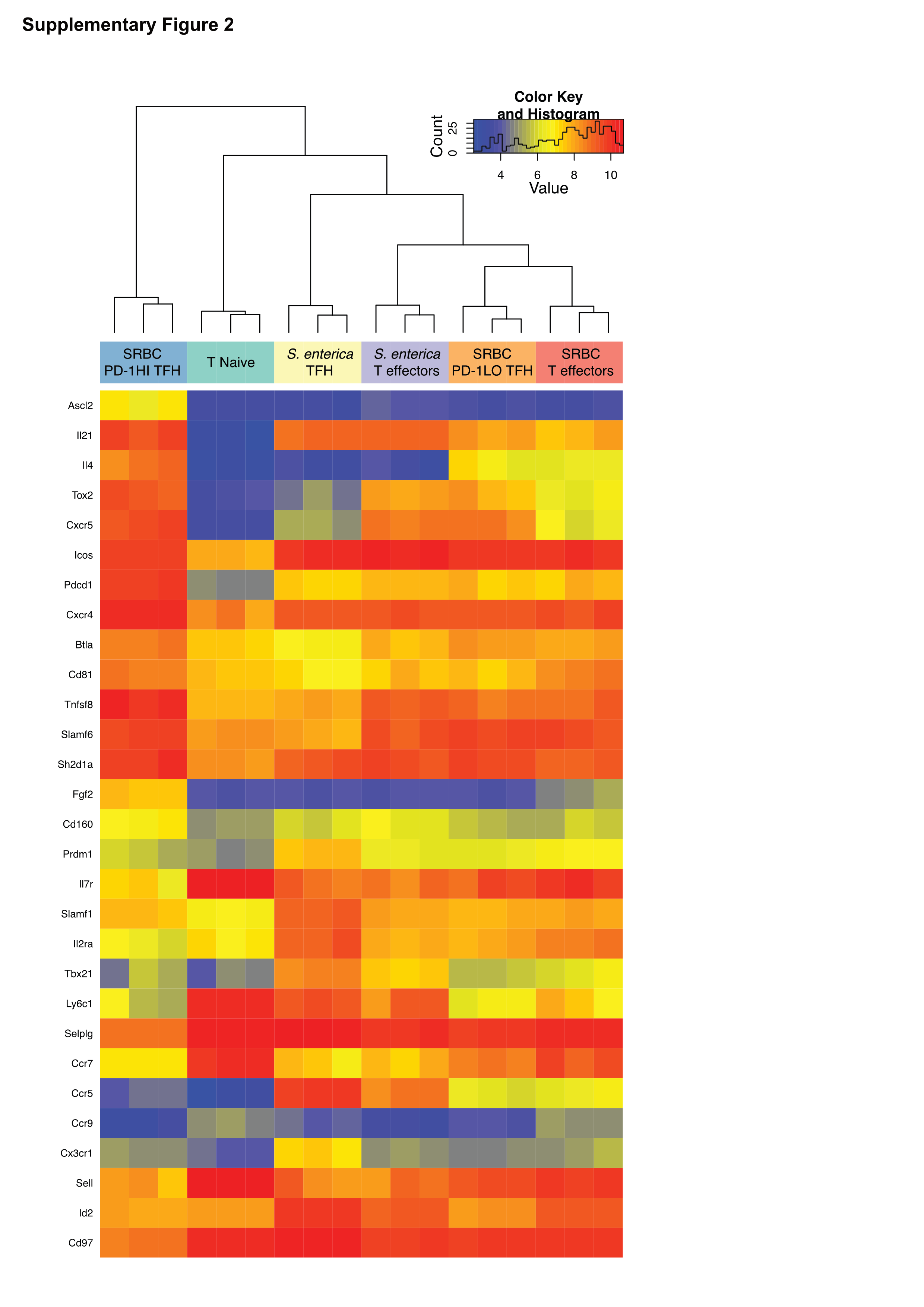

Supplement: Figure S2 — Transcriptional profile analysis of T follicular helper cell (Tfh) subsets by Affymetrix microarray. T cell subsets were isolated by FACS as described in the online methods, and extracted RNA was subjected to Affymetrix microarrays (Mouse Gene 1.1 ST). Data were processed for quality control with arrayQualityMetrics package in Bioconductor and subsequent normalization was achieved using robust multi-array average expression measure. Samples showing similar profiles across analyzed genes are placed next to each other. Heat map represents expression of the transcripts relevant for effector/memory T cell generation. Colors show sample intensities with blue being the lowest (downregulated genes) and red the highest (strongly upregulated genes). Yellow and orange progressively signal intermediate levels of gene upregulation. [file Image_2.TIFF]
